# EHMTI-0179. Emotive and behavior problems in adolescents with cronic headache (CDH) and medication overuse (MOH) using child behaviour checklist(CBCL) e youth self-report (YSR)

**DOI:** 10.1186/1129-2377-15-S1-D40

**Published:** 2014-09-18

**Authors:** MM Mensi, E Maffioletti, E Antonaci, MA Chiappedi, F Ferro, S Molteni, F Galli, F Piazza, U Balottin

**Affiliations:** 1 Child Neuropsychiatry Unit, C. Mondino National Neurological Institute, Pavia, Italy; 2 Child Neuropsychiatry Unit, University of Pavia Pavia Italy, Pavia, Italy

## Introduction

Many studies have examined the importance of psychological factors for the etiopathogenesis of headache in children and adolescents. However, it is not clearly understood whether different types of headache are related or comorbid with specific psychopathological traits.

## Aims

To address this issue, the aim of our study was to investigate the presence of psychological characteristics or peculiar psychopathological traits in subjects with chronic headache and/or medication overuse.

## Methods

Fifty six patients ages 11.0-18.0 (39,3% male and 60,7% female), seen for headache in a third-level centre in Italy, and their 56 mothers were enrolled in this study. They were assessed using Parent Child Behaviour Checklist and Youth Self-report. A detailed history was taken to assess the presence of headache, using criteria defined by ICDH-III beta; in this way we have found: 7 patients with MOH and 24 patients with CDH.

## Results

Patients with CDH at the YSR obtained higher T scores in Thought Problems: this result remained significant both in a three group comparison between patients with probable CDH, patients with confirmed CDH and patients with a non-chronic headache (p=0.04) and in a 2-groups comparison of patients with CDH (both probable or confirmed) or with non-chronic headache (p=0.01). Moreover, we observed a significant reduction of Total Competence score in patients with medication overuse (p=0.02).

## Conclusions

This study confirmed that CDH could be related with a more severe psychopathology, and especially with Thoughts Problems at YSR. Moreover, patients with MOH are described by their mothers as having a reduction of functioning.

No conflict of interest.

